# The impact of breastfeeding on childhood obesity in children that were large-for-gestational age: retrospective study from birth to 4 years

**DOI:** 10.1038/s41598-022-08275-0

**Published:** 2022-03-10

**Authors:** Yinling Chen, Lili Han, Weijuan Su, Ting Wu, Fuping Lyu, Zheng Chen, Bingkun Huang, Liying Wang, Haiqu Song, Xiulin Shi, Xuejun Li

**Affiliations:** 1 Department of Endocrinology and Diabetes, The First Affiliated Hospital of Xiamen University, School of Medicine, Xiamen University, Xiamen, China; 2 Xiamen Clinical Medical Center for Endocrine and Metabolic Diseases, Xiamen, China; 3 Fujian Province Key Laboratory of Diabetes Translational Medicine, Xiamen, China; 4 Fujian Medical University, Fuzhou, China

**Keywords:** Endocrinology, Risk factors

## Abstract

Our aim was to assess effects of breast-feeding (BF) in the association between large-for-gestational age (LGA) and body mass index (BMI) trajectories on childhood overweight from 1 to 4 years old. A total of 1649 healthcare records of mother–child pairs had detailed records of feeding practices and were included in this retrospective cohort study. Data were available in Medical Birth Registry of Xiamen between January 2011 and March 2018. Linear and logistic regression models were used to access the difference between BF and no-BF group. For offspring were LGA and BF was significantly associated with a lower BMI Z-score from 1 to 4 years old after adjustment confounders in Model 1 to 3 [difference in BMI Z-score in Model 1: estimated β: −0.07 [95%CI: −0.13 to −0.01]; Model 2: estimated β: −0.07 (−0.13 to −0.004); Model 3: estimated β: −0.06 (−0.12 to −0.001); *P* = 0.0221, 0.0371, 0.0471]. A significantly lower risk of childhood overweight was observed in Model 1 [odd ratio (OR): 0.85 (95%CI, 0.73 to 1.00)], *P* = 0.0475) with adjustment for maternal pre-pregnancy BMI. Furthermore, Model 2 and Model 3 showed LGA-BF infants had a lower risk for childhood overweight then LGA-no-BF infants [OR: 0.87 and 0.87 (95%CI, 0.73 to 1.03; 0.74 to 1.03)], however, there was no statistical significance (*P* = 0.1099, and 0.1125)]. BF is inversely related to BMI Z-score and risk for overweight in children were LGA from 1 to 4 years old. Adjustment for maternal pre-pregnancy BMI, the protective association between BF and childhood overweight was more significant.

## Introduction

The prevalence of obesity in children have markedly increased in most countries over the past four decades^1^. Children overweight and obesity prevalence has increased from less than 3% in 1985 to 20.5% in 2014^2^. The causes of childhood obesity are multifactorial, such as sedentary behavior, changes in lifestyle, excessive energy intake^3^. Studies indicate that pre-pregnancy body mass index (BMI) is established risk factors for having large-for-gestational age (LGA) infants^4–6^. Meanwhile, LGA infants are more likely to be overweight or obese in childhood^7^.

On the other hand, some studies have shown that obesogenic environment from birth to 2 years old can affect infant development resulting in a higher risk of obesity^8,9^. Obesity developed during this period can enhance risk of excess weight gain over one’s lifetime^10^. Additionally, a study revealed that 11.7% of the children were obese by formula-feeding before 2 years of age compared to 5.6% of children were breast-feeding (BF)^11^. As well, a meta-analysis demonstrated that the children taken in BF were 15% less likely to become overweight as compared to non-BF, like formula-feeding and mixed-feeding^12^.

Furthermore, studies detecting above associations have evaluated child’s BMI, but single BMI cannot account for changes in BMI. A study showed that BMI Z-score trajectory overcomes this limitation, and can be compared throughout ages^13^. Besides, the National Institutes of Health highlighted the exploration of these associations as a major research direction^14^. Thus, the aim of this study was to further confirm if children’s BMI Z-score trajectory and childhood overweight from 1 to 4 years of age is associated with infant feeding practices (BF), based on all children were LGA status, in a longitudinal study of Chinese children.

## Materials and methods

### Study design and study population

We conducted a population-based retrospective study using the healthcare records data were from the Medical Birth Registry in Xiamen system, China, between January 2011 and March 2018. This was a registration system established in 2007 in Xiamen based on a compulsory notification of all live and stillbirths from 12 weeks’ gestation onward. This study was approved by the ethics committee of the First Affiliated Hospital of Xiamen University (KYH2018-007) and conducted in accordance with the rules of the Declaration of Helsinki of 1975, revised in 2013.

A total of 1649 mother–child pair healthcare records were available. All women over the age of 18 performed a 75-g oral glucose tolerance test (OGTT) between 24 and 28 weeks of gestation. We excluded the mother–child pairs with lacking mother’s weight or height data, missing children’s birth weight data, multiple births, premature infants, gestational age < 37 weeks, medical history of diabetes, and fasting glucose level ≥ 7.0 mmol/L before 12 gestational weeks due to the likelihood of having underdiagnosed diabetes pre-pregnancy. The inclusion criteria were as follows: an OGTT was performed between 24 and 28 weeks of gestation; gestational age ate delivery ≥ 37 weeks, with no major neonatal malformations or fetal/neonatal death; and the offspring was followed-up through 4 years of age.

### Data source and linkage

The data source and linkage were referred as our previous published article^4^. All women in Xiamen are registered at their community health centers when they get pregnant, and were then referred to a secondary hospital or a tertiary hospital for healthcare from the 32nd gestational week till delivery. All children were given health examinations at birth (< 3 days afterbirth), and annual examination starting at age 1 until age 4. The data was available for 4 years. Women and children were linked by individual record linkages to the Xiamen citizenhealth information system using the person-unique identification number assigned to each Xiamen citizen. Every child was also linked to his/her biological mother’s maternal identification number.

### Child feeding practices

BF was referred as exclusive breastfeeding in this study. The definitions referred to World Health Organization^15^ as follows: BF while giving no other food or liquid, not even water, with the exception of drops or syrups consisting of vitamins, mineral supplements or medicines. It is measured by following open-ended questions to the parents: ‘For how many months did you breast-feed him/her?’ and ‘How old was your child when you began feeding him/her formula?’^16^.

### Variables definition

#### Large for gestational age

LGA was defined as birth weight was above 90 percentile for gestational age, according to gestational age and gender-specific intergrowth-21st curves^17^.

#### Gestational diabetes mellitus

Gestational diabetes mellitus (GDM) cases were identified by conducting OGTT at 24–28 weeks’ gestation. According to the 2014 National Health and Family Planning Commission of the People’s Republic of China criteria, after a 75 g glucose load, pregnant women would be considered to have GDM if one of the following plasma glucose values was met or exceeded: 0 h, 5.1 mmol/L; 1 h, 10.0 mmol/L; or 2 h, 8.5 mmol/L^18^.

#### Maternal pre-pregnancy BMI classification

Mother’s height and weight were measured in light indoor clothing and without shoes by trained practitioners in community health centers using standardized protocol during pregnancy. Maternal pre-pregnancy BMI was calculated by medical records of self-reported height and weight. BMI was calculated as weight (kg)/height^2^ (m^2^). Referring to WHO criteria^19^, the pre-pregnancy BMI was classified into four groups as follows: underweight (< 18.5 kg/m^2^), normal (18.5–24.9 kg/m^2^), overweight (25.0–29.9 kg/m^2^), and obese (≥ 30.0 kg/m^2^).

### Other maternal and offspring covariates

The maternal and offspring covariates quote from our previous study^4^. Maternal and offspring information were from the Medical Birth Registry in Xiamen system, which included maternal information such as maternal age, education, weight of pre-pregnancy, occupation, first visit date, numbers of pregnancy/infants, last menstrual period, expected delivery date, smoking habits, alcohol use, medical history of diseases, family history of diseases, hypertension history, pregnancy reactions, and labour status. Furthermore, GDM, gestational weight gain, gestational age at delivery, height, weight, blood pressure, fasting glucose, gynaecological examinations, ultrasonography, gestational diabetes screening results, other lab tests results, complications during pregnancy, and pregnancy outcomes were also included in the Medical Birth Registry in Xiamen system. Medical Birth Registry in Xiamen system also included information from newborns to preschool-aged children on date of birth, sex, gestational age at birth, weight, Apgar score, names of the child and his/her parents, family history of diseases, date of examination, weight, height, number of teeth, and blood pressure. Meanwhile, this study is a longitudinal study, attrition were inevitable as more waves of data were contained.

### Offspring measurements and data transformations

During health examination, children’s height and weight was measured by a trained clinician. Before 2 years old, we measured children’s height as length and as standing height to the nearest 0.1 cm after age 2 measured a wall-mounted stadiometer. Body weight was measured in kilograms using regularly calibrated electronic scales. Children were classified into two groups according to LGA and feeding practices status: children born to LGA-BF and children born to LGA-no-BF. BMI Z-score for age was used to present the trajectory tracking of offspring BMI. We calculated age-adjusted z-score of childhood BMI referred to Chinese reference growth charts^20^. Childhood overweight was defined as a BMI at or above the 85th percentile and below the 95th percentile, and obesity was defined as a BMI at or above the 95th percentile^20^. Birth weight was transformed into a Z-score^21^.

### Informed consent

The ethics committee of the First Affiliated Hospital of Xiamen University (KYH2018-007) approved of the waiver for the informed consent because this was a retrospective study.

### Statistical analysis

Mean [standard deviation (SD)] are showed for continuous variables, n (%) were presented as and categorical variables. To evaluate our hypothesis that LGA and BF were associated with offspring BMI growth trajectories, we performed several analyses. Mean BMI Z-score were visually compared in yearly time intervals between LGA-BF and LGA-no-BF. We used linear and logistic regression models to assess the differences in offspring’s anthropometric outcome (BMI Z-score, and overweight/obese) in yearly time intervals between infants that were breastfed as compared to those who were not breastfed. Between BF exposure and anthropometric outcomes measured from 1 to 4 years of age were performed through mixed-effects regression models with random intercepts, accounting for the correlation between repeated observations within subjects. Three multivariable-adjusted models were performed: Model 1 adjusted for maternal pre-pregnancy BMI (continuous); Model 2 was further adjusted for Model 1 + maternal age before pregnancy (continuous), sex (discontinuous), education (high school and above, middle school and below); and Model 3 was adjusted for Model 2 + GDM (yes/no). In addition, we evaluated potential effect modification by modeling the cross-product term of the stratifying variable with LGA-BF. Statistical significance was two-tailed with a *P*-value < 0.05. All calculations were carried out using SAS 9.4 (SAS Institute Inc, Cary, North Carolina, USA).

### Statement of ethics

This study was approved by the ethics committee of the First Affiliated Hospital of Xiamen University (KYH2018-007) and conducted in accordance with the rules of the Declaration of Helsinki of 1975, revised in 2013. Informed consent was not required because this was a retrospective study.

## Results

**Figure 1:**
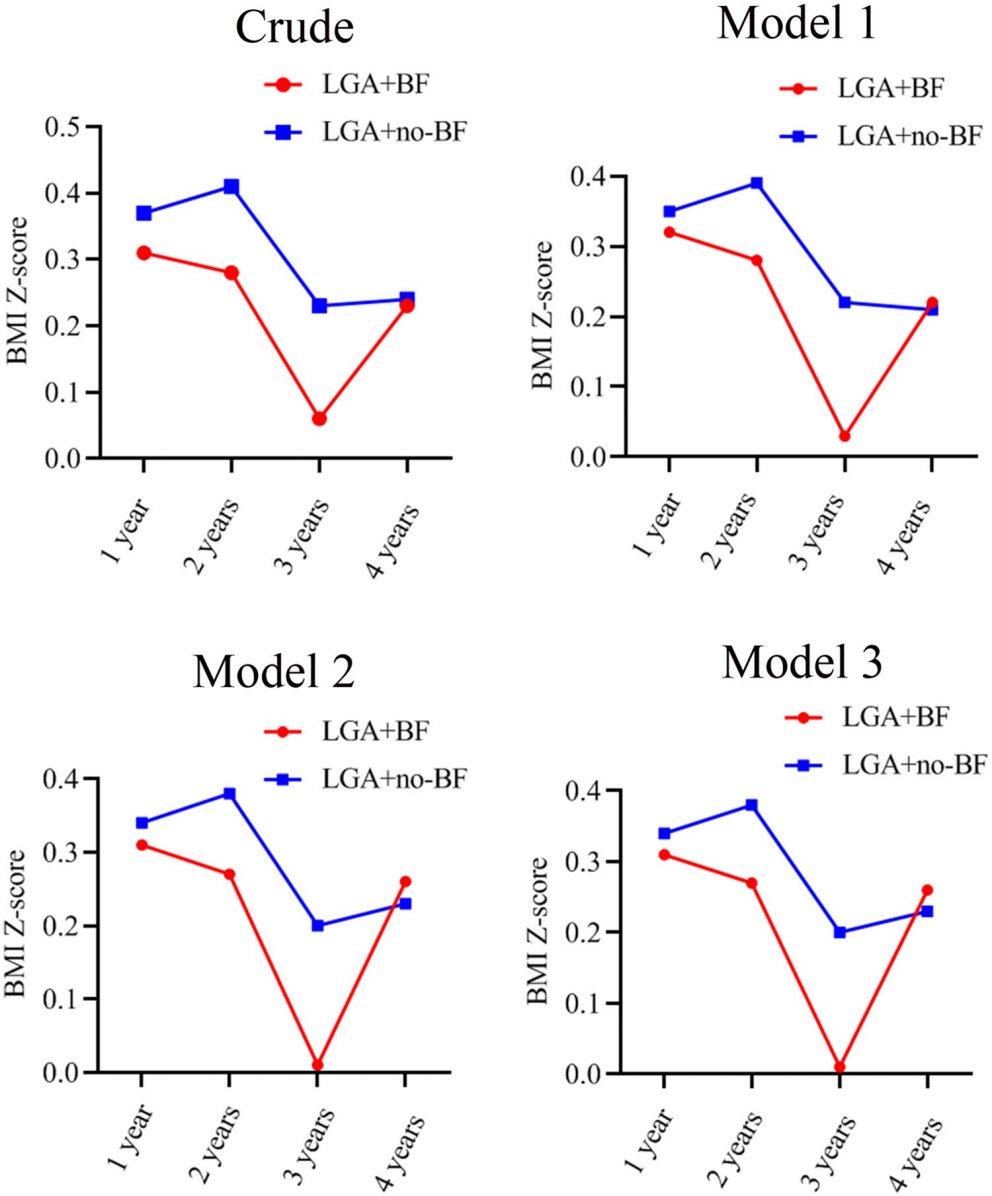
BMI Z-score trajectories of children were LGA-BF and LGA-no-BF from 1 to 4 years in different models. *LGA* large-for-gestation age, *BMI* body mass index, *BF* breast-feeding. Model 1, adjusted for maternal pre-pregnancy BMI; Model 2, adjusted for covariates in Model 1 + maternal age, education, and child sex; Model 3, adjusted for covariates in Model 2 + gestational diabetes melitus.

A total of 1649 mother–child pair healthcare records were available, including 676 children exposed to LGA-BF and 973 offspring exposed to LGA-no-BF. 1613 children were detailedly recorded food practices status at 1 year of age, 646 offspring with BF, 967 children with formula-feeding (FF) or mixed-feeding. Characteristics of participants based on BF status are shown in Table 1. Furthermore, we found that no-BF offspring had a higher BMI Z-score at 3 years of age; however, this relationship was not statistically significant (*P* > 0.05). Additionally, women were in the LGA-no-BF group had slightly higher 1-h and 2-h plasma glucose (*P* < 0.01). The characteristics of mothers and their offspring were similar between the two groups (Table 1). In order to assess BMI Z-score trajectory of offspring who were LGA-BF from 1 to 4 years of age, we adjusted some variates, as showed in Table 2. In model 1, 2, and 3, BMI Z-score trajectory of offspring were LGA-BF without significant difference at 1 year, 2 years, and 4 years of age (all *P* > 0.05). Interestingly, only at 3 years of age, a higher BMI Z-score trajectory presented to LGA-no-BF group in any model (all *P* < 0.05). The specific tendency chart shown in Fig. 1.

**Table 1:** Characteristics of offspring were large-for-gestational-age and breast-feeding.

	Available, n	Child exposed to LGA, and BF (n = 676)	Child exposed to LGA, and no-BF (n = 973)	*P* value
**Maternal characteristics**				
Maternal age before pregnancy, mean (SD), y	1635	28.76 (4.25)	29.17 (4.4)	0.2901
Gestational age at delivery, mean (SD), week	1648	38.67 (1.38)	38.57 (1.37)	0.7794
Pre-pregnancy BMI, mean (SD), kg/m^2^	1649	22.03 (2.81)	21.99 (3.16)	0.0011
**Pre-pregnancy BMI category, n (%)**	1649			0.0376
< 18.5 kg/m^2^	174	62 (3.76)	112 (6.79)	
18.5–24.9 kg/m^2^	1236	528 (32.02)	708 (42.94)	
25.0–29.9 kg/m^2^	212	80 (4.85)	132 (8.00)	
≥ 30.0 kg/m^2^	27	6 (0.36)	21 (1.27)	
Gestational weight gain, mean (SD), kg	159	1.62 (1.79)	1.74 (2.06)	0.3232
**Gestational diabetes mellitus**	1649			0.3574
Yes, n (%)	365	142 (8.61)	223 (13.52)	
No, n (%)	1284	534 (32.38)	750 (45.48)	
**Education, year**	1483			0.7315
≤ 9, n (%)	382	153 (10.32)	229 (15.44)	
> 9, n (%)	1101	452 (30.48)	649 (43.76)	
Mean systolic pressure, mean (SD), mmHg	907	107.0 (11.22)	106.8 (10.75)	0.3649
Mean diastolic pressure, mean (SD), mmHg	906	64.79 (7.82)	65.22 (8.05)	0.5500
Fasting plasma glucose, mean (SD), mmol/L	1649	4.59 (0.41)	4.61 (0.48)	< 0.001
1-h plasma glucose, mean (SD), mmol/L	1649	7.95 (1.59)	8.15 (1.78)	0.0020
2-h plasma glucose, mean (SD), mmol/L	1649	6.80 (1.31)	6.97 (1.57)	< 0.001
**Child characteristics**				
Child sex	1649			0.0092
Boy (%)	1088	455 (67.3)	621 (63.8)	
Girl (%)	561	221 (32.7)	352 (36.2)	
Birth weight, kg	1649	3.7 (0.3)	3.7 (0.3)	0.4000
**BMI Z-scores for age, mean (SD)**				
12 months	1613	0.31 (0.93)	0.37 (0.94)	0.6189
24 months	1157	0.28 (0.83)	0.41 (0.94)	**0.0075**
3 years	843	0.06 (0.93)	0.23 (1.00)	0.1349
4 years	423	0.23 (0.90)	0.24 (0.96)	0.4673

*LGA* large-for-gestational age, *BF* breast-feeding, *BMI* body mass index, *SD* standard deviation.

**Table 2:** Comparison of offspring BMI Z-scores according to offspring were breast-feeding and large-for-gestational age for different age.

BMI Z-score for age	Exposed to LGA and BF	Exposed to LGA and no-BF	*P* value
**Age 1 year**			
Model 1	0.32 (0.04)	0.35 (0.03)	0.5573
Model 2	0.31 (0.04)	0.34 (0.03)	0.5990
Model 3	0.31 (0.04)	0.34 (0.03)	0.5818
**Age 2 year**			
Model 1	0.28 (0.04)	0.39 (0.04)	0.0559
Model 2	0.27 (0.05)	0.38 (0.04)	0.0630
Model 3	0.27 (0.05)	0.38 (0.04)	0.0647
**Age 3 year**			
Model 1	0.03 (0.06)	0.22 (0.05)	**0.0098**
Model 2	0.01 (0.06)	0.20 (0.05)	**0.0122**
Model 3	0.01 (0.06)	0.20 (0.05)	**0.0131**
**Age 4 year**			
Model 1	0.22 (0.09)	0.21 (0.06)	0.8903
Model 2	0.26 (0.10)	0.23 (0.07)	0.7736
Model 3	0.26 (0.10)	0.23 (0.07)	0.7537

Data are showed as mean (SE).

*BMI* body mass index, *LGA* large-for-gestational age, *BF* breast-feeding. Model 1: adjusted for pre-pregnancy BMI; Model 2: adjusted for covariates in Model 1 + child sex, maternal age, and education; Model 3: adjusted for covariates in Model 2 + gestational diabetes mellitus.

In the longitudinal study (Table 3), we found a significant association between LGA-BF and offspring BMI Z-score. LGA-BF offspring had a significantly lower BMI Z-score across the 4 years after adjusted covariates in Model 2 and Model 3 (difference in BMI Z-score estimated β: −0.07 and −0.06 [95% conference interval (CI), −0.13 to −0.004; −0.12 to −0.001], *P* = 0.0371; 0.0471) and a lower trend risk of overweight but not statistically significant (odds ratio (OR):0.87 and 0.87 [95%CI, 0.73 to 1.03; 0.74 to 1.03], *P* = 0.1099; 0.1125). However, adjustment for pre-pregnancy BMI in Model 1, offspring were BF was related with a lower BMI Z-score at 1 year, 2 years, 3 years, and 4 years of age (difference in BMI Z-score: 0.07 [95%CI, −0.13 to −0.01]; *P* = 0.0221) and a lower risk trend for overweight with statistically significant (OR, 0.854 [95%CI, 0.73 to 1.00]; *P* = 0.0475).

**Table 3:** Longitudinal analysis of anthropometric assessed during study visits from 1 to 4 years of age in offspring were large-for-gestational age and breast-feeding.

Outcome	BMI Z-score		Overweight	
	Absolute change in Z-score Estimate β (95% CI)	*P* value	OR (95% CI)	*P* value
Model 1	−0.07 (−0.13 to −0.01)	**0.0221**	0.85 (0.73 to 1.00)	**0.0475**
Model 2	−0.07 (−0.13 to −0.004)	**0.0371**	0.87 (0.73 to 1.03)	0.1099
Model 3	−0.06 (−0.12 to −0.001)	**0.0471**	0.87 (0.74 to 1.03)	0.1125

*LGA* large-for-gestational age, *BMI* body mass index, *CI* confidence interval, *OR* odds ratio. Model 1: adjusted for maternal pre-pregnancy BMI; Model 2: adjusted for covariates in Model 1 + maternal age, education, and child sex; Model 3: adjusted for covariates in Model 2 + gestational diabetes mellitus.

## Discussion

The aim of this population-based study was to assess the relative contribution of LGA and BF on the risk of being overweight in offspring. In a cohort of 1,649 children, the mean BMI Z-score was higher for offspring were LGA-no-BF aged 1 years, 3 years, and 4 years (unadjusted covariates). Additionally, the BMI Z-score of children were LGA-BF was only significant lower at 3 years old in adjusted models. Furthermore, the longitude study indicated that a significant lower risk for BMI Z-score difference of offspring who were LGA-BF from 1 to 4 years old. Offspring were LGA-BF had a significantly low risk trend for overweight in Model 1 with adjustment for maternal pre-pregnancy BMI. However, this association was no significance in Model 2 and Model 3 with adjustment for maternal pre-pregnancy BMI + other covariates.

Since 2007, China has adopted more breastfeeding interventions to promote breastfeeding. Maternity leave has been increased to 180 days in recent years. A meta-analysis^22^ showed that “any breastfeeding” rates are from 63.41 to 92.93% at six months among 2007 and 2018. Meanwhile, the “exclusive breastfeeding” rates at six months were between 17.87 and 58.50%. “any breastfeeding” and “exclusive breastfeeding” rate are all improved in the most recent years.

The estimates in the current study revealed that LGA-BF reduced the risk of childhood overweight by 15.0% compared with LGA-no-BF. A meta-analysis comparing BF with FF infants found a 15% decrease in the odds of childhood overweight^12^. The American Academy of Pediatrics (AAP) stated in 2012 that ‘although complex factors confound studies of obesity, there is a 15–30% reduction in adolescent and adult obesity rates if any BF occurred in infancy compared with non-BF’^23^. Meanwhile, some studies showed that BF is a significant protective factor against obesity in offspring^24,25^. However, this remains controversial. Some studies revealed there was no association between BF and childhood obesity ^26,27^. The reason why there were different results may be attributed to the limited number of included participants, neither this study nor above two studies did a subgroup analysis for feeding duration.

On the other hand, the AAP revealed any BF reduced the risk of otitis media by 23% and of gastroenteritis by 64% ^23^, further, exclusive BF for > 6 months reduced the risk of upper respiratory tract infection by 63%^23^. One study showed BF had higher cognitive function and better scores throughout childhood and adolescence compared with no-BF^28^. The Dutch medical institutions found a ‘protective effect of exclusive BF on asthma, atopic dermatitis, and wheezing in early childhood for at least 4 months^29^. Besides, independent studies have shown that FF also leads to higher morbidity and mortality in western countries^30,31^.

Possible mechanisms linking BF to lower risk for childhood overweight or obesity are not well demonstrated. Breast milk included some bioactive components, such as leptin, adiponectin, insulin-like growth factor-1, and ghrelin, may play a protective role in future offspring overweight or obesity^32,33^. In addition. The quantity of energy and protein in breast milk is lower than that in formula feeding, and the elevated protein intake of formula in infancy would result in rapid growth of adipose^34,35^. A longitudinal study indicated a significant relation between early protein intake and later BMI, showing that children have a higher protein intake early may enhance the risk of later overweight or obesity^36^. Meanwhile, animal researches were also found that protein intake played a key role in body composition and glucose metabolism in later life^37,38^. These findings might provide plausible explanations for a protective role of BF against overweight or obesity.

Interestingly, we found that the BMI Z-score of children were LGA-BF was significant lower at 3 years old with adjustment for GDM. This phenomen may be contributed to maternal GDM. Our previous study^4^ eluciated that GDM have effect on child overweight or obestiy. Meanwhile, maternal pre-pregnancy BMI may play a key role in childhood overweight for children were LGA-BF. Many studies found that pre-pregnancy BMI was significant for later childhood overweight or obesity^39–41^. Furthermore, our another study was also indicated that the association between offspring were LGA and born to maternal GDM and childhood overweight was attenuated after adjusting for pre-pregnancy BMI^4^.

Our study is contemporary, population-based, and results are therefore generalizable. However, the current study still has several limitations. First, this study is a longitudinal study, attrition was inevitable as more waves of data was contained. Second, this study lacked of data on child BMI and BF for all children. Third, reference to measurement error, the outcomes measure, child BMI Z-score could not reflect body fat mass, particularly in young children. Fourth, the no-BF data were not classified as categories due to lacking detailed information of nutrition of breastfeeding such as formula and complementary food. Finally, Considering direct benefits of breastfeeding, the effect may be mainly mediated by higher prevalence of other beneficial lifestyle habits among the these families. Although there are some misclassifications, it is likely to be nondifferentiated and makes our outcomes towards to null. Additionally, adjustments were performed for covariates, thus figuring out the effects of other variables in the reported associations.

## Conclusion

This population-based retrospective study shown that BF is inversely related to risk of overweight or obesity for offspring were LGA who aged 1 to 4 years. Additionally, the association was more significant after adjusting maternal pre-pregnancy BMI. These results suggest that it is important to promote and encourage pregnancy women to breastfeed children. Meanwhile, further research should analyze components of breast milk and feeding practices of mothers with LGA. Furthermore, pregnancy women should focus on pre-pregnancy BMI that might have long-term effects on childhood overweight or obesity. It is significant to explore and develop prenatal interventions that highlight childhood overweight or obesity prevention from an early stage.

## Abbreviations

BF: Breast-feeding
FF: Formula-feeding
LGA: Large-for-gestational age
BMI: Body mass index
MBRX: Medical Birth Registry in Xiamen
OGTT: Oral glucose tolerance test
GDM: Gestational diabetes mellitus
AAP: American Academy of Pediatrics
CI: Confidence interval
OR: Odd ratio
SD: Standard deviation
