# Author Correction: The impact of breastfeeding on childhood obesity in children that were large-for-gestational age: retrospective study from birth to 4 years

**DOI:** 10.1038/s41598-022-10363-0

**Published:** 2022-04-19

**Authors:** Yinling Chen, Lili Han, Weijuan Su, Ting Wu, Fuping Lyu, Zheng Chen, Bingkun Huang, Liying Wang, Haiqu Song, Xiulin Shi, Xuejun Li

**Affiliations:** 1 Department of Endocrinology and Diabetes, The First Affiliated Hospital of Xiamen University, School of Medicine, Xiamen University, Xiamen, China; 2 Xiamen Clinical Medical Center for Endocrine and Metabolic Diseases, Xiamen, China; 3 Fujian Province Key Laboratory of Diabetes Translational Medicine, Xiamen, China; 4 Fujian Medical University, Fuzhou, China

Correction to: *Scientific Reports* 10.1038/s41598-022-08275-0, published online 10 March 2022

The original version of this Article contained errors.

The Title,

“Breastfeeding on childhood obesity in children were large-for-gestational age: retrospective study from birth to 4 years”

now reads:

“The impact of breastfeeding on childhood obesity in children that were large-for-gestational age: retrospective study from birth to 4 years”

